# Fully Automated Pulmonary Lobar Segmentation: Influence of Different Prototype Software Programs onto Quantitative Evaluation of Chronic Obstructive Lung Disease

**DOI:** 10.1371/journal.pone.0151498

**Published:** 2016-03-30

**Authors:** Hyun-ju Lim, Oliver Weinheimer, Mark O. Wielpütz, Julien Dinkel, Thomas Hielscher, Daniela Gompelmann, Hans-Ulrich Kauczor, Claus Peter Heussel

**Affiliations:** 1 Department of Diagnostic and Interventional Radiology, University Hospital of Heidelberg, Im Neuenheimer Feld 110, 69120, Heidelberg, Germany; 2 Department of Diagnostic and Interventional Radiology with Nuclear Medicine, Thoraxklinik at University of Heidelberg, Amalienstrasse 5, 69126, Heidelberg, Germany; 3 Translational Lung Research Center Heidelberg (TLRC), Member of the German Center for Lung Research (DZL), Im Neuenheimer Feld 430, 69120, Heidelberg, Germany; 4 Department of Radiology, German Cancer Research Center (dkfz), Im Neuenheimer Feld 280, 69120, Heidelberg, Germany; 5 Institute for Clinical Radiology, University Hospital, Ludwig-Maximilians University, Munich, Marchioninistr. 15, D-81377, Muenchen, Germany; 6 Division of Biostatistics, German Cancer Research Center (dkfz), Im Neuenheimer Feld 280, 69120, Heidelberg, Germany; 7 Department of Pneumology and Respiratory Critical Care Medicine, Thoraxklinik at University of Heidelberg, Amalienstr. 5, 69126, Heidelberg, Germany; Helmholtz Zentrum München, GERMANY

## Abstract

**Objectives:**

Surgical or bronchoscopic lung volume reduction (BLVR) techniques can be beneficial for heterogeneous emphysema. Post-processing software tools for lobar emphysema quantification are useful for patient and target lobe selection, treatment planning and post-interventional follow-up. We aimed to evaluate the inter-software variability of emphysema quantification using fully automated lobar segmentation prototypes.

**Material and Methods:**

66 patients with moderate to severe COPD who underwent CT for planning of BLVR were included. Emphysema quantification was performed using 2 modified versions of in-house software (without and with prototype advanced lung vessel segmentation; programs 1 [YACTA v.2.3.0.2] and 2 [YACTA v.2.4.3.1]), as well as 1 commercial program 3 [Pulmo3D VA30A_HF2] and 1 pre-commercial prototype 4 [CT COPD ISP ver7.0]). The following parameters were computed for each segmented anatomical lung lobe and the whole lung: lobar volume (LV), mean lobar density (MLD), 15^th^ percentile of lobar density (15^th^), emphysema volume (EV) and emphysema index (EI). Bland-Altman analysis (limits of agreement, LoA) and linear random effects models were used for comparison between the software.

**Results:**

Segmentation using programs 1, 3 and 4 was unsuccessful in 1 (1%), 7 (10%) and 5 (7%) patients, respectively. Program 2 could analyze all datasets. The 53 patients with successful segmentation by all 4 programs were included for further analysis. For LV, program 1 and 4 showed the largest mean difference of 72 ml and the widest LoA of [-356, 499 ml] (*p*<0.05). Program 3 and 4 showed the largest mean difference of 4% and the widest LoA of [-7, 14%] for EI (*p*<0.001).

**Conclusions:**

Only a single software program was able to successfully analyze all scheduled data-sets. Although mean bias of LV and EV were relatively low in lobar quantification, ranges of disagreement were substantial in both of them. For longitudinal emphysema monitoring, not only scanning protocol but also quantification software needs to be kept constant.

## Introduction

Pulmonary emphysema, a phenotype of chronic obstructive pulmonary disease (COPD) induced mostly due to cigarette-smoke, is currently ranked 12^th^ as a worldwide burden of disease and is projected to be ranked 5^th^ by the year of 2020 as a cause of loss of quantity and quality of life [1]. There is currently no definite cure to this major health problem, and many patients remain significantly disabled although evolving pharmacological treatment and pulmonary rehabilitation [2]. Lung volume reduction surgery (LVRS) and bronchoscopic lung volume reduction (BLVR) are strategies in treatment of advanced emphysema. With careful selection of cases, it is reported that LVRS or less invasive BLVR can be clinically beneficial to patients who suffer from heterogeneous advanced emphysema [3–6]. The basic mechanism of these methods is the reduction of hyperinflation of the target lobe, which is identified as the most diseased lobe in case of heterogeneous distribution of emphysema on chest computed tomography (CT) [7]. Expansion of healthier adjacent lung parenchyma and improvement of overall lung function follow.

Complementary to clinical and pulmonary function testing (PFT) [8–10], quantitative multi-detector computed tomography (MDCT) densitometry allows to evaluate the distribution of emphysema (i.e., lobar-based volume and attenuation changes), which can be useful not only for patient selection in treatment planning but also post-interventional follow-up [11–13]. State-of-the art emphysema quantification software programs are being developed, and different programs and/or versions have been implemented into clinical trials or routine care at different institutions. Potential variations in the results of emphysema quantification obtained from those different programs even for the same patient, and the resulting differences in interpretation may pose a source of substantial differences in patient selection and management. The variation of fully automated densitometry results of the whole lung with different software tools was reported recently [14]. Selection for LVRS and BLVR, however, depends mainly on heterogeneity of emphysema and the definition of a target lobe with highest emphysema severity [15]. Most recent tools allow for a lobe-based quantification of emphysema for optimal target lobe definition. This introduces a novel reader-independent further step of lung lobe segmentation into quantitative MDCT accompanied by potential sources of error.

Consequently, we aimed to evaluate the inter-software variability of lobe-based emphysema quantification using 2 versions of scientific software, 1 commercially available software and a pre-commercial prototype for fully automated pulmonary lobar segmentation. We additionally hypothesized and tried to prove that high intra-patient variability of emphysema distribution (one of the requirements for volume reduction surgery or BLVR, as mentioned before) may also predispose for a higher inter-software measurement variability.

## Materials and Methods

### Study population

This study retrospectively enrolled consecutively chronic obstructive pulmonary disease (COPD) patients who underwent clinically indicated MDCT for planning of endoscopic lung volume reduction from August to October 2012. Informed written consent for examination and pseudonymized data processing was obtained from all patients. The responsible Heidelberg University Ethics Committee has approved this study according to Good Clinical Practice (GCP) guidelines and applicable law (S-609/2012). Patients who had pneumothorax at the time of CT scan and history of previous lung operation and those with severe artifacts derived from poor respiratory control at CT were not eligible. Table 1 shows a summary of the patients´ clinical characteristics. There was one never-smoker and smoking history was unknown in one of the patients with smoking history.

**Table 1:** Patient characteristics.

Number of subjects	53
Age (years)	63±7 (range 47–78)
Male/female	31/22
Weight (kg)	75±19
BMI (kg/m^2^)	26±5
Pack years	56±32(range 12–150)
GOLD II/III/IV	2/21/30
VC max (%)	69±19
FEV1(%)	32±8
FEV1/VC	49±11
TLC (%)	133±20
RV (%)	247±57

BMI = body mass index, VC max = maximal vital capacity, FEV1 = forced expiratory volume in 1 s, TLC = total lung capacity, RV = residual volume. Percentage values refer to the predicted volumes.

### MDCT acquisition and reconstruction

Non-enhanced, thin-section MDCT was performed in supine position as recommended for COPD [16, 17]. All patients were trained for full-inspiration and were carefully monitored for inspiration level to be stabilized at full-inspiration before the start of MDCT scanning (64-slice Somatom Definition AS64, Siemens Medical Solutions AG, Forchheim, Germany). The system underwent dedicated routine calibration for water every 3 months and for air daily. We used a dose-modulated protocol using a reference of 120 kV, 70 mA or 100 kV, 117 mA with automated kV and mA modulation (Caredose4D, Siemens Medical Solutions, Forchheim, Germany), collimation of 64 x 0.6 mm, pitch of 1.45, reconstruction slice thickness of 1.0mm with 0.825 mm increment, and medium soft B40f algorithm considered optimal for densitometry and automatic segmentation [10, 18].

### Quantitative image evaluation

MDCT datasets were subjected to the following 4 software programs for fully automated lobar emphysema quantification. No manual correction for segmentation results was carried out. The results were compared for inter-software reproducibility. The processing was repeated after one week for each case and software program in order to evaluate the intra-software reproducibility.

A reader with more than 6 years of experience (HL) performed a visual inspection of the segmentation results for each case and software in order to identify obvious errors on lobar segmentation (e.g. false annotation of a lobe, false identification of a fissure, leakage of airway segmentation into the parenchyma and vice versa). The comparison of the measurement results between programs was thus repeated with the remaining datasets after removal of those with obvious segmentation errors in at least one of the programs for inter-software reproducibility after user-interaction.

#### YACTA

Two versions of in-house program YACTA (“yet another CT analyzer”) (version v.2.3.0.2 and v.2.4.3.1, both programmed by O. W.) applying algorithms without and with advanced lung vessel segmentation were used in this study (program 1 and 2, respectively). The program analyzed each stack of around 300 images per patient fully automatically, as employed in previous studies [10, 19–21]. YACTA operates in a server-mode and may receive DICOM data directly from the PACS. The exact steps of lung and airway segmentation, and emphysema quantification were performed as described in detail elsewhere [22, 23]. When the density of lung voxel was equaled or below the threshold of -950 HU (which is the most often used value currently), it was assigned to emphysema [18, 24], noise correction was performed for voxels with -910 to -949 HU which needed at least 4 adjacent voxels with a density of ≤-950 HU to be annotated as emphysema. The following variables were computed and exported as a structured report: total lung volume (LV) and respective lobar volume (LV_LUL,_ LV_LLL,_ LV_RUL,_ LV_RML,_ LV_RLL_) of the lung, EV, EI, MLD, and 15^th^. In Program 2 (YACTA v.2.4.3.1), an additional algorithm was introduced for an advanced lobe segmentation. While program 1 did assess bronchial tree only into account for lobe separation, program 2 did include the pulmonary vessels additionally.

#### Pulmo3D

Syngo.Via (Pulmo3Dversion VA30A_HF2, Siemens Medical Solutions, Forchheim, Germany) is a commercial post-processing software environment for routine diagnostics (referred to as program 3 in the following). The MDCT datasets were sent from the PACS to the respective post-processing server. The emphysema threshold of -950 HU was chosen as for the other software programs. The parameters measured were: LV, MLD, 15^th^, full width at half maximum of lung density histogram (FWHM), and low attenuation volume in percent (equals EI of other programs). The EV needed to be calculated manually by multiplying low attenuation volume in percent with lung volume.

#### CT COPD

CT COPD is a pre-commercial prototype visualization software package (ISP ver7.0, Philips, Boston, MA) (referred to as program 4 in the following). The DICOM data of each patient was loaded manually into the software surface on a dedicated workstation. A pre-selection of the emphysema threshold is possible, and -950 HU was used for the present study as for the other software programs. The following parameters were calculated by program 4: LV, 15^th^, MLD, EV and EI.

### Pulmonary Function Testing

Whole-body plethysmography (MasterScreen Body, E. Jaeger, Hoechberg, Germany) was performed for each patient within one week prior referral to MDCT [25], and the European Coal and Steal Community (ECSC) predicted values served as the standard of reference [26]. The following lung function parameters (absolute and percent predicted values) were used for further analysis: forced expiratory volume in 1s (FEV1), vital capacity (VC), FEV1 to VC ratio (FEV1/VC, “Tiffeneau index”), residual volume (RV), total lung capacity (TLC). To estimate the degree of hyperinflation, the RV to TLC ratio was calculated (RV/TLC).

### Statistical Analysis

Before exerting statistical analysis, the results from 4 programs were reviewed by a reader with more than 6 years of expertise in chest radiology. None of the software program developers participated in statistical data analysis or interpretation, to allow a fair comparison of all the softwares analyzed. Statistics were done by an independent professional statistician (T.H.). O.W. (the programmer of YACTA) did not participate in data analysis or interpretation in order to allow an un-biased comparison between all programs. Technical replicates per patient from two pairs sessions were averaged. Values were summarized as mean/median and standard deviation/mean absolute deviation by lobes and in total. Spearman's correlation coefficient based on aggregated measurements over all individual lobes per patient was calculated (For spearman’s correlation analysis, repeated measurements due to multiple lobe measurements per patients could not be accounted for in a sensible manner. Lobe measurements were aggregated into a single value per patient. Total measurements refer to the sum across lobes for volume parameters and the average for the other parameters.). Agreement between programs was analyzed for each parameter (LV, MLD, 15^th^, EI and EV) separately, employing Bland-Altman plots and 95% limits of Agreement (LoA). LoA were based on all individual lobes using a random effects model with by segment linked replicates [27]. Pairwise differences in measurements between methods were tested based on a linear random effects model with additional fixed segment effect and random patient effect. EI values were logit transformed prior to testing (Emphysema index is given as percentage which in generally cannot not be considered to be a normal distributed variable. Logit transformation is a standard approach for percentage values–or any variable with values in the range of 0 to 1—to get more normal-like distributed values. Since we used a linear regression approach to test for difference, this transformation was advisable).

To see whether there is an influence of intra-patient variability of EI on inter-program variability, we first assessed intra-patient variability (standard deviation) of EI among lobes for each software. Then, we divided patients into two groups to analyze the effect of intra-patient variability of EI on LOA: patients with low intra-patient variability and those with high intra-patient variability by using the median value of SD. The ratio of predicted SD of LOAs between patients with low and high intra-patient variability was acquired for each pair of programs to see the difference of inter-program variability.

P-values for all pair-wise comparisons were multiplicity adjusted. All tests were two-sided. P-values below 0.05 were considered statistically significant. Statistical analyses were performed using R program [28] with add-on packages MethComp [29], nlme [28] and multcomp [30].

## Results

### Data processing

66 patients with advanced COPD were included initially and all 4 software programs successfully loaded all datasets in DICOM format into their respective servers. Complete unsuccessful segmentation using programs 1, 3 and 4 occurred in 1 (1%), 7 (10%) and 5 (7%) patients, respectively.

**Fig 1:**
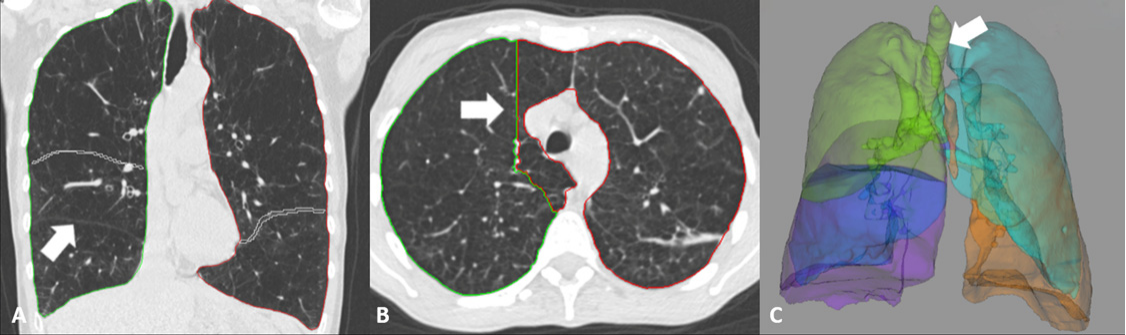
Examples of patients who were excluded. Patients for whom at least one of the programs could not generate results at all were excluded from the analysis. (A) Program 3 could not recognize right middle lobe (arrow) in this 57 year-old patient with FEV1 = 24% due to unsuccessful lobar segmentation. (B) A 50 year-old patient with FEV1 = 20% had incorrect outline of the lung and could not be processed normally in program 3. (C) Program 4 included part of the central airway to right upper lobe (arrow) in this 75 year-old patient with FEV1 = 44%. Program 3 also failed in lobar segmentation for the same patient. FEV1 = forced expiratory volume in 1s.

**Fig 2:**
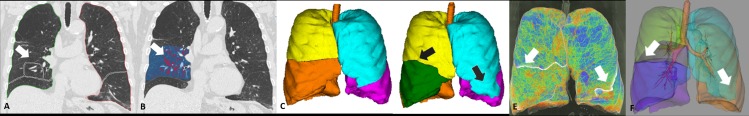
Examples of patients who were included. Our main purpose was to evaluate fully automated emphysema quantification. Therefore, some patients who had substantially different values by different software programs were included for analysis before user interaction. (A, B) A 72 year-old patient with FEV1 = 51% had incomplete minor fissure (arrow). LV for RML was 87 ml by using program 3 (A) and 718 ml using program 4 (B). (C-F) In this 66 year-old patient with FEV1 = 26%, RML could not be delineated by program 1 (C, RML LV = 0.465 ml). However, LV of RML was 976 ml by using program 2 (D), which uses additional algorithm to that of program 1 and this result was similar to those of program 3 (E, RML LV = 906 ml) and 4 (F, RML LV = 934 ml). There was also improvement in left upper lobe segmentation by using program 2 compared to the result by program 1 (arrows). LV = lobar volume, RML = right middle lobe

Program 1 failed to segment the right upper lobe for one patient in both of the sessions. The segmentation failed because the right upper lobe bronchi were not segmented by the bronchial tree segmentation algorithm causing the following lobe segmentation algorithm to fail also. There was an unexpected halt with program 3 during the lobar segmentation process of 6 patients (Fig 1A), and erroneous outline of the lung was produced in another patient (Fig 1B). Program 4 also failed to generate results during the segmentation process of 5 patients. In one patient in which segmentation could not be achieved with program 3, program 4 also delivered erroneous results due to segmentation of part of the central airway as right upper lobe (Fig 1C). Program 2 was able to analyze all datasets. We observed that right upper lobe and right middle lobe were major source of relative variability of lobar segmentation resulting in difference of quantification results considering the course of the minor fissure. Fig 2 shows the example of the patients who had substantially different values by programs.

Excluding image date retrieval, pure computational runtime was around 3–4 minutes for all programs. The values from the 53 patients who were successfully assessed with all programs were used for further analysis (for software variability using lobe-by-lobe basis).

### Intra-software reproducibility

There was 100% reproducibility for each value between 2 paired sessions of program 1, 2 and 3. In program 4, the values based on the total lung were the same between 2 sessions. However, minimal discrepancies of lobar-based results occurred. The mean difference was almost negligible with a mean difference in two sessions of 0.063 ml for lobar volume, 0.3 HU for MLD, 0.011 HU for 15^th^, 0.057 ml for EV and 0.05% for EI (using lobe-by-lobe basis as mentioned above).

### Inter-software variability of fully automated analysis

The means of measurements from each program are shown in Table 2. The results of Bland-Altman analysis for each parameter are summarized in Table 3. According to the pairwise tests on difference, LV was significantly different between program 1 and 4, and 2 and 4 (*p* = 0.02, both). Program 1 and 4 showed the largest mean difference of 72 ml and the widest limits of agreement (LoA) of [-356, 499ml].

**Table 2:** Overview of the densitometry results (n = 53).

Median Values (mean absolute deviation)		Program 1	Program 2	Program 3	Program 4
**LV [ml]**	**RUL**	1505 (341)	1629 (489)	1408 (426)	1410 (258)
	**RML**	520 (205)	472 (115)	511 (178)	587 (248)
	**RLL**	1634 (352)	1637 (385)	1501 (489)	1583 (376)
	**LUL**	1722 (390)	1642 (378)	1551 (404)	1534 (402)
	**LLL**	1574 (303)	1574 (337)	1587 (278)	1487 (343)
	**Total**	7068 (1403)	7068 (1404)	6845 (1379)	6722 (1348)
**MLD [HU]**	**RUL**	-877 (24)	-875 (24)	-899 (22)	-905 (22)
	**RML**	-865 (20)	-863 (25)	-888 (22)	-893 (22)
	**RLL**	-859 (31)	-856 (30)	-880 (29)	-885 (29)
	**LUL**	-867 (16)	-867 (17)	-891(16)	-896 (17)
	**LLL**	-859 (28)	-862 (28)	-887 (27)	-891 (27)
	**Total**	-865 (19)	-864 (19)	-888 (16)	-894 (14)
**15**^**th**^ **percentile of lung density [HU]**	**RUL**	-971 (18)	-971 (18)	-979 (16)	-971 (17)
	**RML**	-959 (14)	-961 (15)	-966 (13)	-956 (14)
	**RLL**	-961 (18)	-959 (18)	-970 (16)	-958 (19)
	**LUL**	-965 (14)	-964 (15)	-971 (12)	-963 (13)
	**LLL**	-962 (21)	-963 (19)	-973 (18)	-960 (17)
	**Total**	-962 (11)	-963(11)	-973 (10)	-960 (13)
**EI [%]**	**RUL**	379 (22)	37 (21)	38 (17)	36 (22)
	**RML**	25 (14)	26 (12)	27 (9)	20 (10)
	**RLL**	24 (14)	25 (16)	26 (14)	21 (13)
	**LUL**	30 (11)	29 (12)	29 (10)	25 (11)
	**LLL**	29 (15)	30 (15)	31 (13)	24 (13)
	**Total**	32 (11)	32 (11)	32 (9)	29 (10)
**EV [ml]**	**RUL**	581 (371)	570 (353)	532 (298)	425 (281)
	**RML**	118 (87)	107 (83)	129 (54)	102 (50)
	**RLL**	466 (259)	436 (307)	421 (226)	356 (272)
	**LUL**	492 (178)	521 (239)	454 (216)	347 (169)
	**LLL**	371 (205)	387 (221)	407 (196)	340 (182)
	**Total**	2188 (1015)	2189 (1016)	2084 (816)	1819 (962)

LV = lung volume, EV = emphysema volume, EI = emphysema index, MLD = mean lung density, HU = Hounsfield units, RUL = right upper lobe, RML = right middle lobe, RLL = right lower lobe, LUL = left upper lobe, LLL = left lower lobe.

**Table 3:** Variation of densitometry (n = 53).

	Programs	1–2	1–3	1–4	2–3	2–4	3–4
**LV (ml)**	**r**	1	1	1	1	1	1
	**Mean difference (**Δ)	0.	61	72	61	72	10
	**Limits of agreement**	-418, 418	-359, 481	-356, 499	-344, 457	-331, 475	-395, 416
**EV (ml)**	**r**	1	1	1	1	1	1
	**Mean difference (**Δ)	0	10	61	10	61	51
	**Limits of agreement**	-162, 162	-177, 198	-109, 232	-181, 202	-113, 236	-148, 250
**EI (%)**	**r**	1	1	1	1	1	1
	**Mean difference (**Δ)	0	0	3	0	3	4
	**Limits of agreement**	-6, 6	-8, 7	-6, 12	-8, 7	-6, 12	-7, 14
**MLD (HU)**	**r**	1	1	1	1	1	1
	**Mean difference (**Δ)	0	25	30	24	29	5
	**Limits of agreement**	-37, 28	6, 44	10, 49	1, 48	6, 53	-8, 18
**15**^**th**^ **percentile of lung density (HU)**	**r**	1	1	1	1	1	1
	**Mean difference (**Δ)	0	8	-1	8	-1	-9
	**Limits of agreement**	-7, 8	-3, 19	-12, 10	-3, 18	-12, 10	-22, 4

LV = lung volume, EV = emphysema volume, EI = emphysema index, MLD = mean lung density, HU = Hounsfield units. Mean differences (Δ) and limits of agreement were calculated in accordance with the approach of Bland and Altman.

The difference for MLD was significant between program 1 and 3, 1 and 4, 2 and 3, 2 and 4, and 3 and 4 (*p*<0.001 for all except between program 3 and 4, *p* = 0.008 between program 3 and 4). The largest difference for MLD was between program 1 and 4. The LoA was widest between program 2 and 4 for MLD. In Bland-Altman plot describing MLD, 95% confidence interval is narrower between program 3 and 4 than other pairs (data not shown), indicating better agreement between two programs. As for comparing MLD values between program 3 and 1, program 3 and 2, program 4 and 1, and program 4 and 2 (data not shown), 95% confidence interval is below the line of equality, indicating that program 1 and 2 overestimates MLD in all cases relatively to program 3 and 4 (which is connected with the fact that program 1 and 2 calculate greater lung volumes).

In case of 15^th^, there were significant differences between program 1 and 3, 2 and 3, and 3 and 4 (*p*<0.001). As for the 15^th^, the narrowest interval was depicted between program 1 and 2 (data not shown). Program 3 and 4 revealed the largest mean difference and the widest LoA for 15^th^.

As for EV, there were significant differences between program 1 and 4, 2 and 4, and 3 and 4 (*p* = 0.005, 0.005 and 0.02, respectively). The difference for EV was largest between program 1 and 4 with a mean difference of 61 ml. However, the widest LoA existed between program 3 and 4 [-148, 250 ml].

There were significant differences for EI between program 1 and 4, 2 and 4, and 3 and 4 (*p* = 0.003, 0.003 and <0.001. respectively). Program 3 and 4 showed the largest mean difference of 4% and the widest LoA of [-7, 14%] for EI.

### Influence of intra-patient variability

The median standard deviation (inter-quartile range) of the EI amongst the lobes of each single patient as a marker of intra-patient variability was 9.86% (7.67–13.24) for program 1, 9.86% (7.11–13.38) for program 2, 8.99% (5.85–12.16) for program 3, and 9.67% (7.60–13.72) for program 4. The pairwise correlation of intra-patient variability between software pairs ranged from 0.95 (program 1 vs. program 4) to 1 (program 1 vs. program 2). We then used the median SD of the intra-patient EI to separate patients into groups with low and high intra-patient EI variability. Interestingly, the group with high intra-patient variability also showed wider LAO for inter-software variability oft he EI, which was up to 1.81 times higher than in the group with low intra-patient variability (Table 4). This effect was not dependent on the software used for determining intra-patient EI variability (data not shown).

**Table 4:** Predicted standard deviation (SD) of Limits-of-agreement (LAO) for emphysema index (EI) for the inter-software comparison grouped for patients with low and high intra-patient variability of the EI.

Programs	Low intra-patient variability	High-intra-patient variability	ratio
1–2	2.55	2.93	1.15
1–3	3.24	4.46	1.37
1–4	3.17	5.73	1.81
2–3	3.27	4.12	1.26
2–4	3.19	5.48	1.72
3–4	3.77	6.42	1.71

Median SD of the intra-patient variability of EI determined with program 2 served as a cut-off for group definition.

### Influence of user interaction

After visual inspection by a thoracic radiologist considerable errors in lobar segmentation were found in 27 of 53 patients: program 1: 11 patients, program 2: 9 patients, program 3: 2 patients, program 4: 3 patients, both program 1 and 4: 2 patients). Notice that the datasets where the programs 1, 3 and 4 delivered complete unsuccessful segmentations (1, 7 and 4 datasets, respectively) were not contained in these 27 datasets.

The comparison of the measurement results between programs was thus repeated with the remaining 26 datasets (S1 Table and S2 Table) after removal of those with obvious segmentation errors in at least one of the programs for inter-software reproducibility after user-interaction.

The LoA between the software tools for LV, MLD, 15^th^, EV and EI became smaller after user interaction and removal of all datasets with obvious segmentation errors in one or more software tool, reflecting the improvement of inter-software agreement in the remaining pairs after this interaction (S3 Table). The mean differences, however, did not change substantially for all parameters (S4 Table). Importantly, even after this user interaction densitometric results for MLD, 15^th^ and EI remained significantly different between the programs.

## Discussion

The main findings of the present study about emphysema quantification using fully automated lobar segmentation are as follows: (1) intra-program reproducibility was generally excellent in all four programs in moderate to severe emphysema patients with various degree of destroyed lungs and distorted course of a fissure due to severe emphysematous changes of adjacent lung parenchyma; (2) the mean difference of lobar LV, MLD, 15^th^, EV and EI are very small among different software programs; (3) LoA, however, remains substantially wide, resulting in non-interchangeability of the results obtained from different software programs; (4) high intra-patient variability of EI resulted in a higher inter-software variability of EI.

Compared to PFT or other clinical examinations, one of the main strength of quantitative MDCT in evaluating emphysema lies in offering regional information about the distribution of emphysema from lobar segmentation, which enables selection of the target lobe to be treated regionally e.g. by BLVR [31]. Fully automated lobar segmentation and lobe-based emphysema quantification should be preferred to semi-automated or manual segmentation methods because it is more time efficient especially in the setting of clinical routine practice in specialized centers. The latter two obviously imply inter- and intra-user variability depending on the operators’ skill and perseverance [32]. Also, previous study reported that automated and semi-automated lobar quantification of emphysema are concordant and show good agreement with visual scoring [33]. However, the software used here did rely mainly upon vessel segmentation instead of bronchial course [33], which does result in problems especially in joint vascular regions such as segment 3 as part of upper lobe and segment 4 as part of middle lobe most frequently having common pulmonary vasculature (S1A–S1F Fig) [34].

The clinical relevance of a diagnostic modality depends on its ability to provide reproducible results regardless of the influence of external factors [35]. Along the chain of quantitative MDCT for emphysema, most factors have been previously studied including inspiration depth, radiation exposure parameters, kernels, reconstruction methods and slice thickness [14]. In a recent study, we could show that inter-software variability for whole-lung emphysema quantification is higher than the natural inter-individual variability of emphysema [14]. Since then, novel software has emerged, introducing fully automated lobe segmentation into quantitative MDCT for regional emphysema quantification.

In the present study, we intended to evaluate the impact of selecting different software tools on fully automated lobar emphysema quantification by comparing the results from 4 different software programs. Firstly, even commercial programs were not able to process all provided data successfully. After exclusion of those error inducing data-sets, we found a high degree of correlation and linearity between results derived from the four software programs in our study. However, these high correlation values should not be incorrectly interpreted as a measure of the interchangeability between results from different programs. In Bland-Altman analysis, calculated values are located around the mean line within a 95% confidence interval for perfect agreement [36].

In the present study, we did not observe good agreement between different programs mostly because LoA on Bland-Altman plots were not narrow enough to be considered as negligible from our radiological perspective. In general, program 1 and 2 (which are different version of YACTA basing therefore upon the same start algorithm and applying identical noise-correction) showed better agreement compared with other pairs of software programs. Even between program 1 and 2 which are different version of the same program without and with application of advanced lung vessel segmentation, however, there existed some bias (mean difference) for all of the five parameters. For example, we could notice one extreme example of substantial improvement in LV measurement by program 2 compared with that by program 1, which is probably due to an additional algorithm considering pulmonary vessels (Fig 2C and 2D). Program 2 did improve the lobar segmentation at the level of the smaller broncho-pulmonary bundles, where not all the bronchi might have been segmented. This explains program 2 revealing the smallest LV for middle lobe as compared to all other 3 programs (Table 2).

There are some researchers who believe that EI directly reflects the phenomenon of destruction of lung tissue that occurs in emphysema, diminishing the partial volume effect of air and lung structures on each voxel of the affected zone, unlike percentile lung density [37]. In the current study, the lines of mean difference for EI locate close to the line of equality in every pair of programs (S2 Fig), suggesting that all programs provide similar results. If we take the largest bias of 4% (between program 3 and 4), however, this value is too big considering Harris´ proposal and previously published data [38]. Also, this bias amounted to 13% of the values from global EI measurement in this study. It was reported that the variability of EI measured with two different programs should be approximately less than 1% [14].

According to the previous study [14], potential reasons of error in whole lung emphysema quantification among different software programs are as follows: the steps of lung segmentation (e.g the use of different morphologic algorithms or the the inclusion of leakage from airways into lung parenchyma), airway segmentation (the extent of airway segmentation into the periphery of airway tree) and subsequent emphysema segmentation, and variations in noise correction among software programs.

The additional measurement variation in the present study probably comes from different lobar segmentation algorithms in the current study, resulting in different LV and subsequent different densitometry values. In patients with an inhomogeneous emphysema distribution who are generally more suitable for volume reduction surgery or BLVR, we found a higher inter-software variability, probably related to a stronger distortion of normal lobe anatomy posing additional challenges to lung lobe detection. Thus, patients with inhomogeneous emphysema are prone to a higher measurement variability of computational emphysema quantification.

As we expected, we found that right upper lobe and right middle lobe were major sources of relative variability (considering fissure) due to sharing of the other fissure (minor fissure) by visual observation, in agreement with the previous study [39]. Fissure analysis was not included as a part of algorithm in any of 4 programs that we used. Although fissure analysis might give additional information for lobe separation and more accurate values, it also related to very variable results between subjects, the problem of frequent incompleteness of fissures and confounding factors such as scarring and subsegmental atelectasis near the fissure especially in the target patients. For more accurate lobar segmentation results, it would be ideal to use editing function of the each program by radiologists. However, this would require extra time and endeavor, which prevents it from being widely used in real clinical practice. There is also the problem of objectivity including inter- and intra-observer reproducibility when we use manual or even semi-automated methods. The scope of this study was focused on the evaluation of the software´s current potential in fully automated lung densitometry.

There are several limitations in the current study. First, there is no *in-vivo* gold standard existing for lobar-based emphysema quantification. Measurements of emphysema quantification software programs are usually based on segmentation of airways on MDCT data sets and an algorithm that translates the segmented voxels into lung volumes. Depending on the voxels included in each segmentation, the result of density histograms can be produced in a different way although segmentation volumes are the same or very similar in amount. It is also known that the segmentation of the pulmonary lobes becomes cumbersome when the inter-lobar boundary is unclear [40], which might cause further problem of accuracy among software programs. Therefore, the visual assessment performed by an experienced reader frequently serves as a “silver-standard”. To learn at least something about the weaknesses of fully-automated lobe segmentation, a second work-up was implemented in patients with limited inter-software match and an experienced reader improve the segmentation algorithms. However, the purpose of this study was not to evaluate accuracy, as this is currently impossible. It was rather to compare the current state of different software programs and see whether it is possible to monitor patients with different programs or to interchange the results among hospitals using different programs. The message is: As CT-equipment and scanning protocols have to be kept constant, also the software used for emphysema quantification has to be the same, although all software programs tested here do deliver a good quality. Second, dose protocol was adapted on the fly individually to the patients’ absorption by using a 4D-care dose technology. Before starting this study, we examined whether there is any significant effect of this on emphysema quantification. We used the same type of GOLD II-IV patients for this and found that there is no impact of two types of protocols after statistical analysis (not shown). Besides many technical parameters in CT-scanning and image reconstruction, radiation exposure is one of factors that have effect on densitometry results [21, 41]. However, the factor of radiation exposure did not influence our analysis of inter-software comparison that included one MDCT examination per patient–we did not find a trend between the slightly different acquisition technologies. The subjects of the current study are composed of moderate to severe degree emphysema patients, who are the main population of interest for BLVR. Thus, the results are valid in this target population. The potential differences of variability induced by the degree of emphysema were not determined.

In conclusion, we should not interpret the results from different software programs as interchangeable. The significant differences between software programs used for lobar emphysema quantification may lead to contradictory target lobe selection for BLVR in some cases. Another important issue is that different emphysema quantification programs or different versions of the same program may be used in different institutions, impairing comparability of study results. When performing follow-up studies in patients, the software tool should be kept exactly constant.

## Supporting Information

S1 Fig An example of association between degree in distortion of the normal lobe anatomy and inhomogeneity in emphysema distribution, and the influence of the distortion on lobe-based quantification of regional pulmonary emphysema. (A and B) Coronal images of a 62 year old patient with FEV1 = 51% demonstrate distorted incomplete right minor fissure and relatively severe emphysematous change in LLL. (C~F) Sagittal images shows suboptimal segmentation of RML and LLL. RML = right middle lobe LLL = left lower lobe
(DOCX)

S2 Fig Interprogram variability. Bland-Altman plots demonstrate inter-program comparison for EI. The central thick line indicated the mean difference and the upper and lower thin lines indicate upper and lower limits of agreement. EI = emphysema index
(DOCX)

S1 Table Patient characteristics after user interaction. (DOCX)

S2 Table Overview of the densitometry results after user interaction. (DOCX)

S3 Table Variation of densitometry after user interaction. (DOCX)

S4 Table Intra-program repeatability in program 4 before and after user interaction. (DOCX)
