# Supplementary material for: Fully Automated Pulmonary Lobar Segmentation: Influence of Different Prototype Software Programs onto Quantitative Evaluation of Chronic Obstructive Lung Disease
Source: PLoS One. 2016 Mar 30;11(3):e0151498. doi: 10.1371/journal.pone.0151498 (PMC4814108; doi:10.1371/journal.pone.0151498)
Supplement: S1 Fig — (A and B) Coronal images of a 62 year old patient with FEV1 = 51% demonstrate distorted incomplete right minor fissure and relatively severe emphysematous change in LLL. (C~F) Sagittal images shows suboptimal segmentation of RML and LLL. RML = right middle lobe LLL = left lower lobe (DOCX) [file pone.0151498.s001.docx]

**Supporting information**

**S1 Fig. An example of association between degree in distortion of the normal lobe anatomy and inhomogeneity in emphysema distribution, and the influence of the distortion on lobe-based quantification of regional pulmonary emphysema.**

(Figure A and B in S1 File) Coronal images of a 62 year old patient with FEV1=51% demonstrate distorted incomplete right minor fissure and relatively severe emphysematous change in LLL. (Figure C~F in S1 File) Sagittal images shows suboptimal segmentation of RML and LLL.

RML=right middle lobe LLL=left lower lobe
