# Supplementary material for: Fully Automated Pulmonary Lobar Segmentation: Influence of Different Prototype Software Programs onto Quantitative Evaluation of Chronic Obstructive Lung Disease
Source: PLoS One. 2016 Mar 30;11(3):e0151498. doi: 10.1371/journal.pone.0151498 (PMC4814108; doi:10.1371/journal.pone.0151498)
Supplement: S2 Fig — Bland-Altman plots demonstrate inter-program comparison for EI. The central thick line indicated the mean difference and the upper and lower thin lines indicate upper and lower limits of agreement. EI = emphysema index (DOCX) [file pone.0151498.s002.docx]

**Supporting information**

**S2 Fig. Interprogram variability.**

Bland-Altman plots demonstrate inter-program comparison for EI. The central thick line indicated the mean difference and the upper and lower thin lines indicate upper and lower limits of agreement.

EI = emphysema index
