# Supplementary material for: Fully Automated Pulmonary Lobar Segmentation: Influence of Different Prototype Software Programs onto Quantitative Evaluation of Chronic Obstructive Lung Disease
Source: PLoS One. 2016 Mar 30;11(3):e0151498. doi: 10.1371/journal.pone.0151498 (PMC4814108; doi:10.1371/journal.pone.0151498)
Supplement: S1 Table — (DOCX) [file pone.0151498.s003.docx]

**Supporting information**

**S1 Table. Patient characteristics after user interaction**

| **Number of subjects** | **26** |
| --- | --- |
| Age (years) | 63±7 |
| Male/female | 15/11 |
| Weight (kg) | 75±19 |
| BMI (kg/m^2^) | 26±5 |
| Pack years | 47±27 |
| GOLD II/III/IV | 1/11/14 |
| VC max (%) | 73±18 |
| FEV1(%) | 33±9 |
| FEV1/VC | 47±48 |
| TLC (%) | 133±19 |
| RV (%) | 242±48 |

BMI=body mass index, VC max=maximal vital capacity, FEV1=forced expiratory volume in 1 s, TLC=total lung capacity, RV=residual volume. Percentage values refer to the predicted volumes.

The patients who had substantially different values by programs were excluded for the analysis after user interaction (n=27).
