# Supplementary material for: Fully Automated Pulmonary Lobar Segmentation: Influence of Different Prototype Software Programs onto Quantitative Evaluation of Chronic Obstructive Lung Disease
Source: PLoS One. 2016 Mar 30;11(3):e0151498. doi: 10.1371/journal.pone.0151498 (PMC4814108; doi:10.1371/journal.pone.0151498)
Supplement: S2 Table — (DOCX) [file pone.0151498.s004.docx]

**Supporting information**

**S2 Table. Overview of the densitometry results after user interaction**

| **Median Values (mean absolute deviation)** |  | **Program 1** | **Program 2** | **Program 3** | **Program 4** |
| --- | --- | --- | --- | --- | --- |
| **LV (ml)** | **RUL** | 1427 (358) | 1413 (481) | 1351 (488) | 1339 (390) |
|  | **RML** | 569 (258) | 469 (181) | 460 (222) | 506(235) |
|  | **RLL** | 1644 (379) | 1662 (362) | 1535 (440) | 1582 (362) |
|  | **LUL** | 1596 (513) | 1589 (470) | 1475 (381) | 1422 (376) |
|  | **LLL** | 1631 (426) | 1648 (415) | 1524 (369) | 1506 (375) |
|  | **Total** | 6756 (1600) | 6755 (1600) | 6530 (1535) | 6423 (1503) |
| **MLD (HU)** | **RUL** | -877 (23) | -874 (23) | -899 (21) | -901 (24) |
|  | **RML** | -861 (22) | -860 (22) | -878 (21) | -885 (21) |
|  | **RLL** | -850 (26) | -851 (26) | -876 (25) | -881 (22) |
|  | **LUL** | -865 (18) | -865 (20) | -889 (18) | -893 (18) |
|  | **LLL** | -855 (27) | -855 (28) | -880 (28) | -885 (28) |
|  | **Total** | -863 (17) | -863 (17) | -885 (13) | -891 (13) |
| **15^th^ percentile of lung density (HU)** | **RUL** | -971 (19) | -971 (19) | -979 (16) | -970 (19) |
|  | **RML** | -955 (18) | -955 (19) | -960 (18) | -953 (16) |
|  | **RLL** | -957 (18) | -957 (19) | -969 (15) | -957 (18) |
|  | **LUL** | -966 (13) | -965 (15) | -972 (13) | -964 (13) |
|  | **LLL** | -961 (18) | -961 (18) | -973 (18) | -960 (18) |
|  | **Total** | -970 (13) | -970 (13) | -977 (10) | -969 (12) |
| **EI (%)** | **RUL** | 39 (19) | 37 (19) | 37 (17) | 34 (22) |
|  | **RML** | 21 (15) | 21 (16) | 21 (13) | 16 (14) |
|  | **RLL** | 22 (15) | 21 (16) | 24 (12) | 20 (14) |
|  | **LUL** | 29 (12) | 28 (13) | 29 (11) | 25 (12) |
|  | **LLL** | 29 (18) | 25 (18) | 25 (17) | 26 (15) |
|  | **Total** | 32 (10) | 32 (10) | 32 (8) | 29 (10) |
| **EV (ml)** | **RUL** | 526 (353) | 493 (382) | 459 (347) | 441 (347) |
|  | **RML** | 117 (99) | 101 (96) | 106 (78) | 77 (85) |
|  | **RLL** | 350(295) | 390 (297) | 360 (273) | 336 (245) |
|  | **LUL** | 436 (251) | 390 (286) | 410 (251) | 335 (210) |
|  | **LLL** | 346 (294) | 342 (293) | 340 (289) | 294 (274) |
|  | **Total** | 2001 (1133) | 2001 (1133) | 1941 (1022) | 1746 (1099) |

LV = lung volume, EV = emphysema volume, EI = emphysema index, MLD = mean lung density, HU = Hounsfield units, RUL=right upper lobe, RML=right middle lobe, RLL=right lower lobe, LUL=left upper lobe, LLL=left lower lobe.

The patients who had substantially different values by programs were excluded for the analysis after user interaction (n=27).
