# Supplementary material for: Fully Automated Pulmonary Lobar Segmentation: Influence of Different Prototype Software Programs onto Quantitative Evaluation of Chronic Obstructive Lung Disease
Source: PLoS One. 2016 Mar 30;11(3):e0151498. doi: 10.1371/journal.pone.0151498 (PMC4814108; doi:10.1371/journal.pone.0151498)
Supplement: S3 Table — (DOCX) [file pone.0151498.s005.docx]

**Supporting information**

**S3 Table. Variation of densitometry after user interaction**

|  | **Programs** | **1-2** | **1-3** | **1-4** | **2-3** | **2-4** | **3-4** |
| --- | --- | --- | --- | --- | --- | --- | --- |
| **LV (ml)** | **r** |  | 1 | 1 | 1 | 1 | 1 |
|  | **Mean difference (**Δ) | 0 | 60 | 71 | 60 | 71 | 11 |
|  | **Limits of agreement** | - 2382, 238 | -174, 295 | -184, 326 | -146, 267 | -159, 300 | -215, 236 |
| **EV (ml)** | **r** | 1 | 1 | 1 | 1 | 1 | 1 |
|  | **Mean difference (**Δ) | 0 | 9 | 56 | 9 | 56 | 47 |
|  | **Limits of agreement** | -91, 91 | -93, 111 | -53, 164 | -82, 99 | -42, 154 | -62, 155 |
| **EI (%)** | **r** | 1 | 1 | 1 | 1 | 1 | 1 |
|  | **Mean difference (**Δ) | 0 | 0 | 3 | 0 | 3 | 3 |
|  | **Limits of agreement** | -5, 5 | -6, 5 | -3, 9 | -7, 6 | -3, 9 | -3, 10 |
| **MLD (HU)** | **r** | 1 | 1 | 1 | 1 | 1 | 1 |
|  | **Mean difference (**Δ) | 0 | 24 | 29 | 24 | 29 | 5 |
|  | **Limits of agreement** | -9, 10 | 14, 33 | 19, 39 | 13, 34 | 17, 40 | -6, 17 |
| **15^th^ percentile of lung density (HU)** | **r** | 1 | 1 | 1 | 1 | 1 | 1 |
|  | **Mean difference (**Δ) | 0 | 8 | -1 | 8 | -1 | -9 |
|  | **Limits of agreement** | -7, 7 | 0, 15 | -8, 6 | -1, 16 | -9, 7 | -17, -1 |

LV = lung volume, EV = emphysema volume, EI = emphysema index, MLD = mean lung density, HU = Hounsfield units. Mean differences (Δ) and limits of agreement were calculated in accordance with the approach of Bland and Altman.
