# Supplementary material for: Fully Automated Pulmonary Lobar Segmentation: Influence of Different Prototype Software Programs onto Quantitative Evaluation of Chronic Obstructive Lung Disease
Source: PLoS One. 2016 Mar 30;11(3):e0151498. doi: 10.1371/journal.pone.0151498 (PMC4814108; doi:10.1371/journal.pone.0151498)
Supplement: S4 Table — (DOCX) [file pone.0151498.s006.docx]

**Supporting information**

**S4 Table. Intra-program repeatability in program 4 before and after user interaction**

|  | **Before user interaction (n=53)** | |  | **After user interaction (n=26)** | |
| --- | --- | --- | --- | --- | --- |
|  | **Mean difference** (Δ) | **Limits of agreement** |  | **Mean difference** (Δ) | **Limits of agreement** |
| **LV (ml)** | 0 | -235, 235 |  | 0 | -18, 19 |
| **MLD (HU)** | 0 | -9, 9 |  | 0 | -7, 6 |
| **15th percentile of lung density (HU)** | 0 | -3. 3 |  | 0 | -2, 2 |
| **EV (ml)** | 0 | -52, 52 |  | 0 | -5, 5 |
| **EI (%)** | 0 | -2, 2 |  | 0 | 0, 0 |

LV = lung volume, EV = emphysema volume, EI = emphysema index, MLD = mean lung density, HU = Hounsfield units. Mean differences (Δ) and limits of agreement were calculated in accordance with the approach of Bland and Altman.

The patients who had substantially different values by programs were excluded for the analysis after user interaction (n=27).
